# Hypoxia induced Bcl-2/Twist1 complex promotes tumor cell invasion in oral squamous cell carcinoma

**DOI:** 10.18632/oncotarget.13890

**Published:** 2016-12-10

**Authors:** Yuansheng Duan, Qinghua He, Kai Yue, Haishan Si, Jiaxin Wang, Xuan Zhou, Xudong Wang

**Affiliations:** ^1^ Department of Maxillofacial and E.N.T Oncology, Tianjin Medical University Cancer Institute and Hospital, Tianjin 300060, China; ^2^ National Clinical Research Center for Cancer, Key Laboratory of Cancer Preventionand Therapy, Tianjin 300060, China

**Keywords:** Bcl-2, Twist1, tumor invasion, EMT, oral squamous cell carcinoma

## Abstract

Bcl-2 and Twist1 can be coactivated by hypoxia in hepatocellular carcinoma to promote tumor cell metastasis and vasculogenic mimicry, but their function in oral squamous cell carcinoma (OSCC) remains undefined. We employed a cohort of 82 cases of OSCC samples to examine the coexpression of Bcl-2 and Twist1 by immunohistochemistry and demonstrate the interaction between Bcl-2 and Twist1 by coimmunoprecipitation. Bcl-2 and Twist1 overexpression was associated with a poor pathological grade and tumor prognosis, and the two factors functions as a complex. Knocking down Bcl-2/Twist1 inhibited cell migration, decreased cell invasion and inversed cell epithelial-mesenchymal transition (EMT) procession. An animal model derived from the Tca8113 cell line was used to further validate the role of Bcl-2/Twist1 depletion in suppressing tumor EMT and growth. In conclusion, Bcl-2/Twist1 complex can be treated as a potential therapeutic target for OSCC.

## INTRODUCTION

Oral squamous cell carcinoma (OSCC) is the most common pathological type of oral cancer, and is the major cancer type for head and neck carcinoma [1]. It is characterized by local invasion and a high rate of cervical lymph nodes metastasis, which are the main factors contributing to poor prognosis. The epithelial-mesenchymal transition (EMT) has generally been considered as an important mechanism for tumor metastasis, which converts epithelial cells into a mesenchymal-like phenotype [2]. This phenotypic switch including loss of cell contact, upregulation of N-cadherin, Vimentin, Snail, Slug, Twist1 and decreased expression of E-cadherin inversely is important for cell for cancer cells to migrate, invade and induce tumor dissemination from primary site to a secondary organ [3, 4].

As an important hallmark of EMT, Twist1, which is a highly conserved basic helix-loop-helix transcriptional factor, is overexpressed in various human solid tumors including breast cancer [5], lung cancer [6], melanoma [7], head and neck cancer [8], esophageal squamous cell carcinoma [9] and glioblastoma [10]. The role of Twist1 in malignant tumors is associated with initiating tumor EMT and facilitating tumor invasion and metastasis. A high Twist1 expression level directly suppresses E-cadherin expression by interacting with its promoter, and it promotes the synthesis of N-cadherin and fibronectin, thereby remodeling the phenotype and enhancing cell motility and tumor progression [11]. Moreover, EMT-induced Twist1 could induce the rapid dissemination of cytokeration-positive epithelial cells and initiate dramatic transcriptional changes in the extracellular compartment and cell-matrix adhesion genes [12]. In the specific tumor environment, Twist1 undergoes relocation into the nucleus to exhibit its transcriptional regulation effect. Twist1 has a nuclear localization signal (NLS). Sun et al. [13] indicated functional and structural interactions between Bcl-2 and the EMT-regulating transcription factor Twist1 and the relationship with metastasis and vascular mimicry. In their study, Bcl-2 can bind to Twist1 and formation of the Bcl-2/Twist1 complex facilitates the nuclear transport of Twist1 and increase the tumor cell plasticity, metastasis, and vasculogenic mimicry.

The B-cell lymphoma-2 (Bcl-2) gene was originally discovered by Yoshide Tsujimoto at the chromosomal breakpoint translocation when chromosome 18 translocated to chromosome 14 in B-cell lymphomas [14], both antiapoptosis members such as BCL-2, BCL-XL, BCL-W, A1, MCL1, and proapoptosis members such as BAK and BAX were discovered [15]. Pro-survival protein Bcl-2 plays role in oncogenesis. It is well known that tumors develops as a result of an imbalance between cell division/proliferation and cell apoptosis/death. Evasion of programmed cell death has been recognized as one of the six essential alterations in cell physiology that dictate malignant growth, and it is a hallmark of most, and maybe all cancer type. Importantly, the pro-survival protein Bcl-2 plays a key role in this process [16]. High Bcl-2 expression was detected in most cancer types, and it confers a poor prognosis due to its antiapoptosis effect. In addition, Bcl-2 family members also interact with other proteins to modify and regulate cellular metabolism, immune response, and autophagy [17]. However, the mechanisms by which Bcl-2 regulates tumor EMT is not well understood.

In the present study, we first analyzed the expression of Bcl-2, Twist1 and EMT-related proteins in OSCC tissue samples, and elucidated the relationship between Bcl-2/Twist1 and EMT in OSCC. Further, the coexpression of Bcl-2/Twist1 and EMT-related proteins was evaluated in OSCC Tca8113 and Tb3.1 cell lines of OSCC. We found that the depletion of Bcl-2/Twist1inhibited OSCC cell migration, invasion and EMT both *in vitro* and in a Tca8113P160 derived tumor model. Our results support that the Bcl-2/Twist1 complex induces EMT and facilitates tumor metastasis in OSCC, which might represent a powerful strategy for developing of novel OSCC therapies.

## RESULTS

### Bcl-2 and Twist1 were simultaneously overexpressed and correlated with OSCC EMT and poor patient prognosis

**Figure 1:**
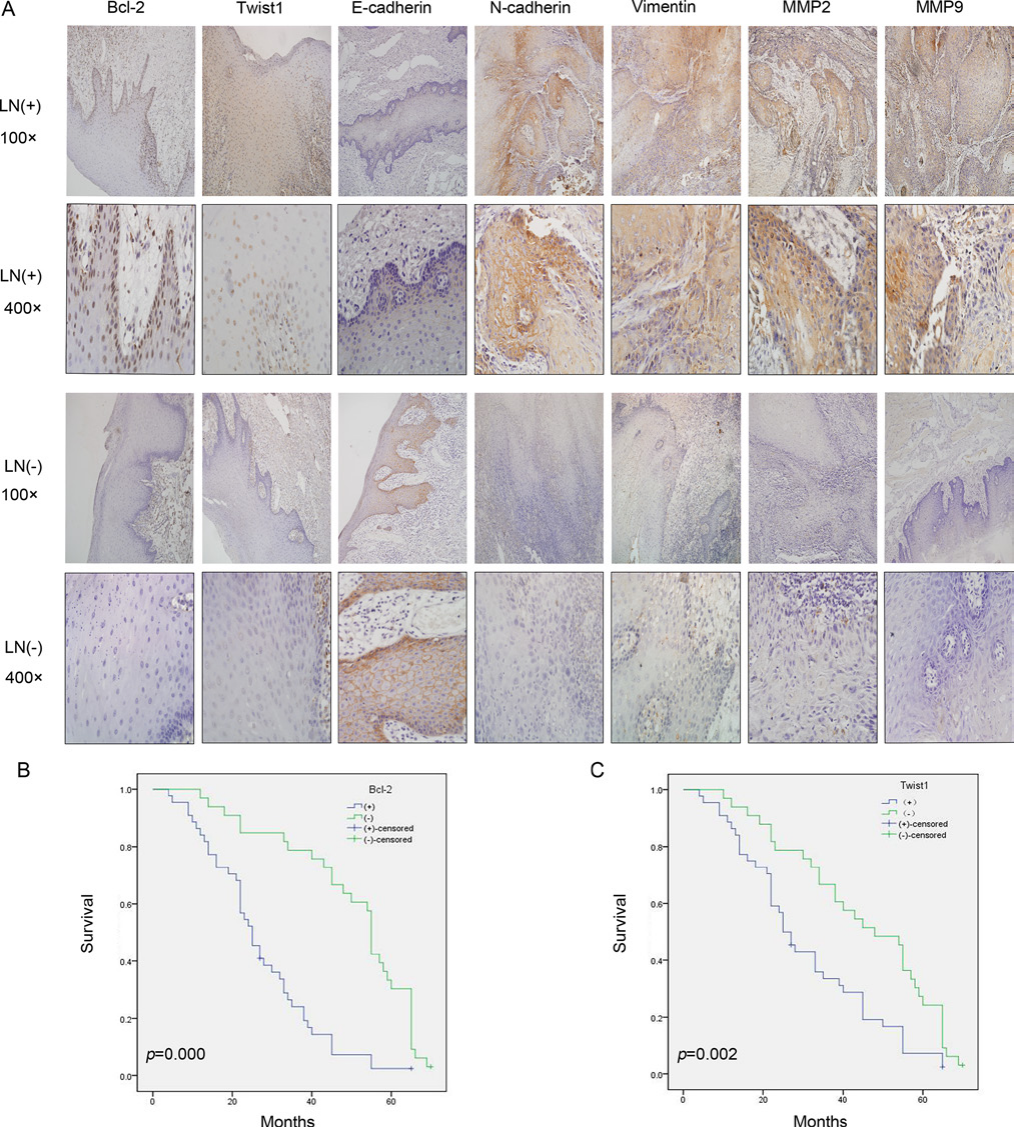
Bcl-2, Twist1 and EMT-related protein expression in the groups with lymph node metastasis (LN+) and non-lymph node metastasis (LN-) and the correlation between Bcl-2 or Twist1 with overall survival **(A)** IHC showed Bcl-2 positive staining was present in the cytoplasm and nucleu, and Twist1 positive staining was observed in the nucleus. Vimentin and N-cadherin positive staining were obvious in the LN (+) group, and they were located in the membrane and cytoplasm. E-cadherin was located in the membrane and had stronger expression in the LN (−) group. MMP-2/9 showed stronger staining in the cytoplasm in the LN (+) group. **(B)** and **(C)** Survival curves for patients with OSCC, in relation to Bcl-2 or Twist1 expression, respectively. The patients with low Bcl-2 and Twist1 expression had a better prognosis.

First, an IHC assay was performed to assess the expression of Bcl-2, Twist1 and EMT-related proteins in 82 OSCC tumor tissues and 24 para-neoplastic tissues. Bcl-2 protein had a high expression level in 48 of the OSCC tissues (58.5%), which was significantly higher than that in the para-neoplastic tissues (8, 33.3%; *P* = 0.005).Twist1 proteins were strongly positive in 46 OSCC tissues (56.1%) and 6 paraneoplastic tissues (25%, *P* = 0.001) (Table 1). Then, the expression levels of EMT-related proteins were examined. The clinicopathological correlations with the expression of the proteins mentioned above are described in Table 2. The expression levels of Bcl-2, Twist1, E-cadherin, N-cadherin, Vimentin and MMP-2/9 were significantly associated with lymph node metastasis (Table 2 and Figure 1A), and the differences in the pathologic grades were significant. The correlation between Twist1 and Bcl-2 and the EMT-related proteins was significant (Table 3). Kaplan-Meier survival analysis suggested that positive Bcl-2 and Twist1 were correlated with poor patient survival (Bcl-2, *p* = 0.000; Twist1 *p* = 0.002). The other indicators were not statistically significant (Figure 1B and 1C).

**Table 1:** Expression of Bcl-2, Twist1 in OSCC

Type	Total	Bcl-2 High	Bcl-2 Low	χ2	P	Twist1 High	Twist1 Low	χ2	P
Tumor	82	48 (58.54%)	34 (41.46%)	8.355	0.005	46 (56.10%)	36 (43.90%)	11.564	0.001
paraneoplastic	24	6 (25.00%)	18 (75.00%)			4 (16.67%)	20 (83.33%)		

**Table 2:** Expression of Bcl-2, Twist1 and EMT related proteins were determined by immunohistochemistry, and clinic-pathologic variables in 82 OSCC

Variables		Twist1				Bcl-2				E-cadherin				N-cadherin				Vimentin				MMP2				MMP9			
	Total	High	Low	χ2	P	High	Low	χ2	P	High	Low	χ2	P	High	Low	χ2	P	High	Low	χ2	P	High	Low	χ2	P	High	Low	χ2	P
Gender																													
Male	56	29	27	2.075	0.150	30	26	1.794	0.180	28	28	0.421	0.636	32	24	1.090	0.339	36	20	0.058	0.811	34	22	2.075	0.150	42	14	1.958	0.162
Female	26	17	9			18	8			15	11			18	8			16	10			20	6			23	13		
Age																													
≤60	49	29	20	0.363	0.547	30	19	0.362	0.547	24	25	0.584	0.503	32	17	0.960	0.363	31	18	0.001	1.000	31	18	0.363	0.547	39	10	0.008	0.930
>60	33	17	16			18	15			19	14			18	15			23	10			23	10			26	7		
T stage																													
T1 & T2	47	24	23	0.844	0.358	26	21	0.470	0.493	24	23	0.083	0.826	28	19	0.091	0.363	29	18	0.139	0.818	29	18	0.844	0.358	34	13	3.216	0.073
T3 & T4	35	22	13			22	13			19	16			22	13			23	12			25	10			31	4		
Lymphnode metastasis																													
Yes	40	29	11	6.950	0.008	29	11	4.228	0.040	16	24	4.845	0.046	29	11	4.359	0.044	31	9	6.678	0.012	32	8	6.950	0.008	37	3	8.320	0.004
No	42	17	25			19	23			27	15			21	21			21	21			22	20			28	14		
Histology grade																													
Grade 1	42	18	24	4.711	0.030	20	22	6.274	0.012	28	14	6.988	0.014	20	22	6.455	0.014	22	20	4.518	0.041	23	19	4.711	0.030	29	13	5.473	0.019
Grade 2 & 3	40	28	12			28	12			15	25			30	10			30	10			31	9			36	4		
Smoking & Drinking																													
Yes	38	22	16	0.000	0.991	23	15	0.116	0.743	22	16	0.845	0.384	22	16	0.282	0.654	27	11	1.781	0.251	25	13	0.000	0.991	28	10	1.344	0.246
No	44	24	20			25	19			21	23			28	16			25	19			29	15			37	7		

**Table 3:** Correlation of Bcl-2, Twist1 and EMT related proteins

	Bcl-2	Twist1	N-cadherin	Vimentin	E-cadherin	MMP2	MMP9
Bcl-2	..	*r* = 0.303	*r* = 0.291	*r* = 0.234	*r* = −0.405	*r* = 0.490	*r* = 0.302
		*P* = 0.006	*P* = 0.008	*P* = 0.034	*P* = 0.000	*P* = 0.000	*P* = 0.006
Twist1		..	*r* = 0.249	*r* = 0.348	*r* = −0.547	*r* = 0.399	*r* = 0.457
			*P* = 0.024	*P* = 0.001	*P* = 0.000	*P* = 0.000	*P* = 0.000
N-cadherin			..	*r* = 0.223	*r* = −0.161	*r* = 0.320	*r* = 0.208
				*P* = 0.044	*P* = 0.148	*P* = 0.003	*P* = 0.061
Vimentin				..	*r* = −0.216	*r* = 0.147	*r* = 0.174
					*P* = 0.051	*P* = 0.187	*P* = 0.119
E-cadherin					..	*r* = −0.325	*r* = −0.306
						*P* = 0.003	*P* = 0.005
MMP2						..	*r* = 0.330
							*P* = 0.002

### Hypoxia enhances Bcl-2 /Twist1 interaction by facilitating Bcl-2 binding toTwist1

**Figure 2:**
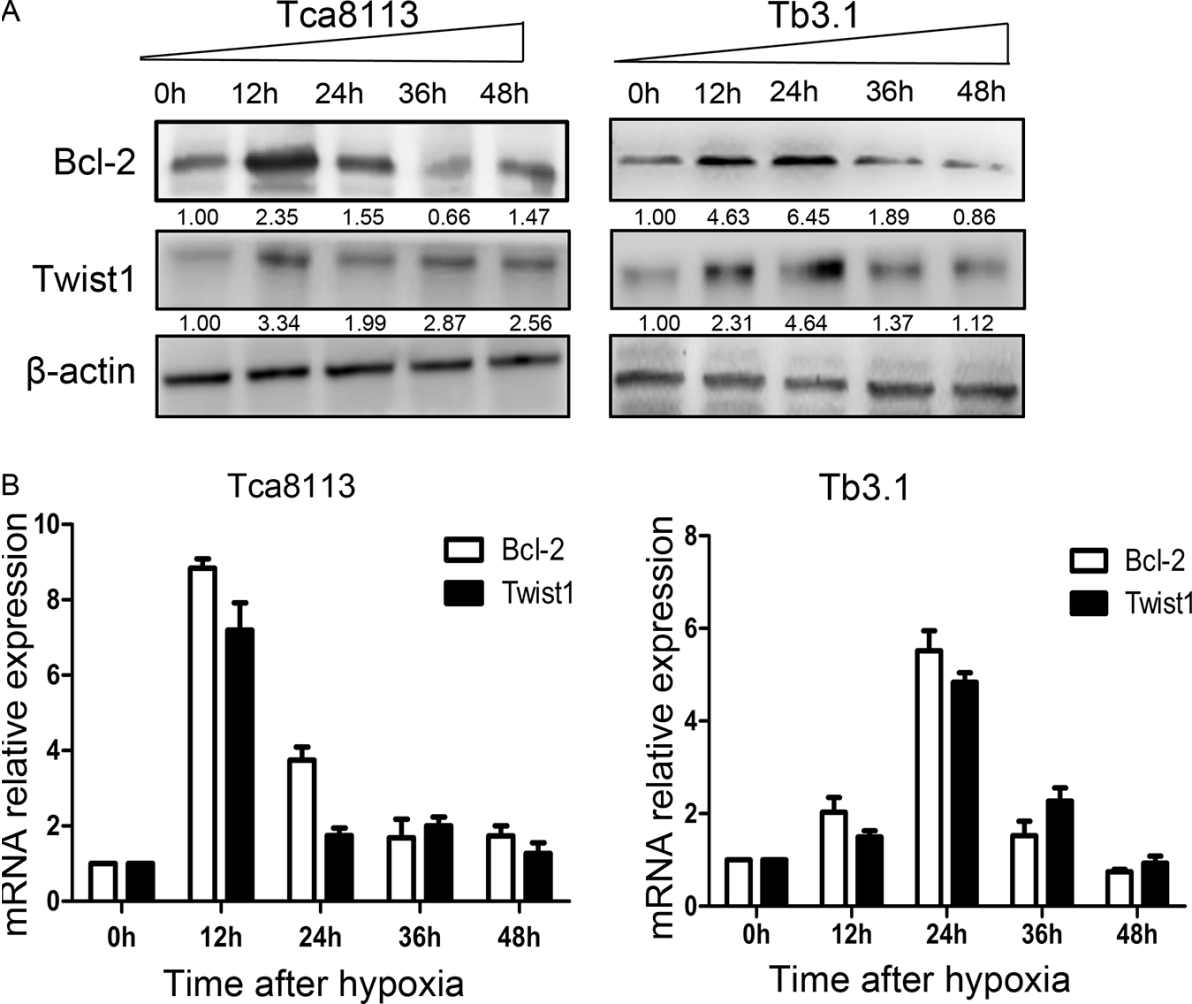
Hypoxia conditions enhance Bcl-2 and Twist1 expression **(A)** Expression levels of Bcl-2 and twist under hypoxia conditions were assessed with western blot analysis. In two cell lines Tca8113 and Tb3.1, Bcl-2 and Twist1 showed similar expression kinetics induced by hypoxia. The expression peaks of Bcl-2 and Twist1 in Tca8113 were detected when hypoxia was relieved after 12 hours, while Tb3.1 peaks at 24 hour. **(B)** For quantitative PCR evaluated messenger RNA (mRNA) , the results were similar.

**Figure 3:**
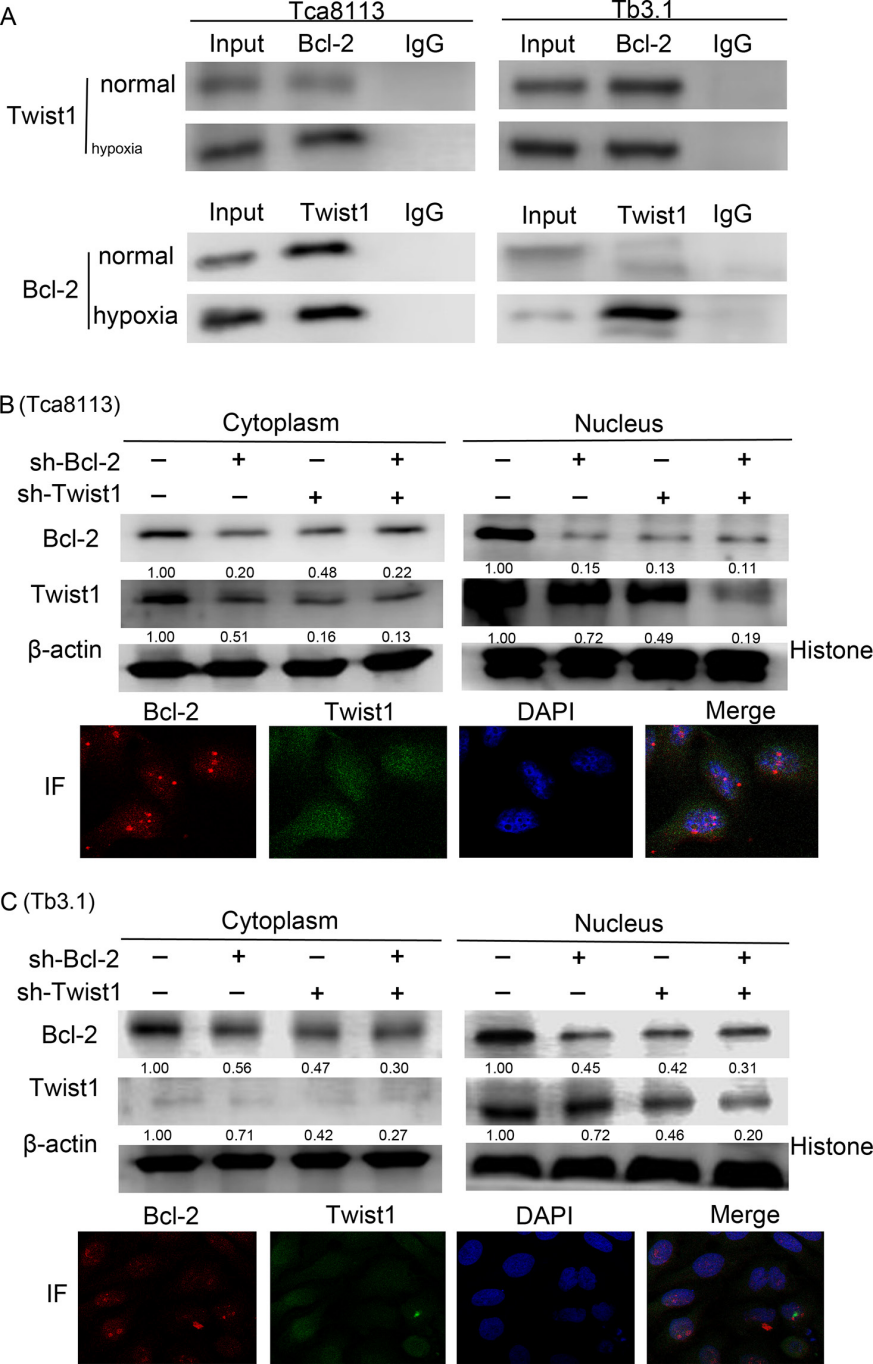
Bcl-2 and Twist1 form a complex in OSCC **(A)** Coimmunoprecipitation was used to determine the interaction of two proteins. Hypoxia treatment was performed in normal Tca8113 cells for 12 hours and Tb3.1 cells for 24 hours. Positive staining could be found in either normal conditions or cell precipitation samples treated with hypoxia in the two cell lines. Hypoxia could boost its interaction. **(B)** and **(C)** Expression of Bcl-2 and Twist1 in the nucleus and cytoplasm following transfection of shRNA against Bcl-2 and Twist1 alone or together in the presence of hypoxia. Immunofluorescence staining examined the dynamic colocalization of these two proteins within single living cells. The Bcl-2 expression is shown in red, whereas Twist1 expression is shown in green. The cells were cultured in hypoxia conditions.

To further explore the correlation and mechanism of interaction between Bcl-2 and Twist1, the Tca8113 and Tb3.1 cell lines were used. CoCl_2_ was used to mimics hypoxia conditions and both Bcl-2 and Twist1 can be induced by hypoxia. Then, hypoxia-induced up-regulation of Bcl-2 and Twist1 was detected after 0, 12, 24, 36 and 48 h of hypoxia by Western blot and quantitative PCR, respectively (Figure 2A and 2B). The mRNA or protein level of Bcl-2 and Twist1 in Tca8113 cells showed expression peaks approximately 12 hours after hypoxia induction and 24 hours in Tb3.1 cells. Two molecules showed similar expression kinetics for each cell line. Additionally, after the upregulation peak, it gradually decreased, which was potentially a result of protein degradation and cell death induced by hypoxia,as described in Sun's research [13]. To further demonstrate the interaction between the proteins, coimmunoprecipitation was used to evaluate the protein complex *in vivo*. As shown in Figure 3A, an antibody against Twist1 coprecipitated the Bcl-2. Similarly, antibody against Bcl-2 coprecipitated Twist1. Furthermore, their binding affinity was enhanced after hypoxia treatment in Tca8113 for 12 hours and Tb3.1 for 24 hours. Next, we performed immunofluorescence staining and examined the dynamic colocalization of Bcl-2 and Twist1 in single living cells. In the nucleus, Bcl-2 has a high expression level compared with that in the cytoplasm and a decrease in Twist1 expression accompanied decreased Bcl-2 levels (Figure 3B and 3C). These results suggest that in OSCC ,Blc-2 and Twist1 form a protein complex and function in synergy in their transportation from the cell cytoplasm to nucleus, as the silence of either Bcl-2 or Twist1 could interfere with complex formation. Additionally, the formation of the Bcl-2/Twist1 complex is stimulated when tumor cells are hypoxic.

### Targeted silence of Bcl-2/Twist1 blocks tumor progression

**Figure 4:**
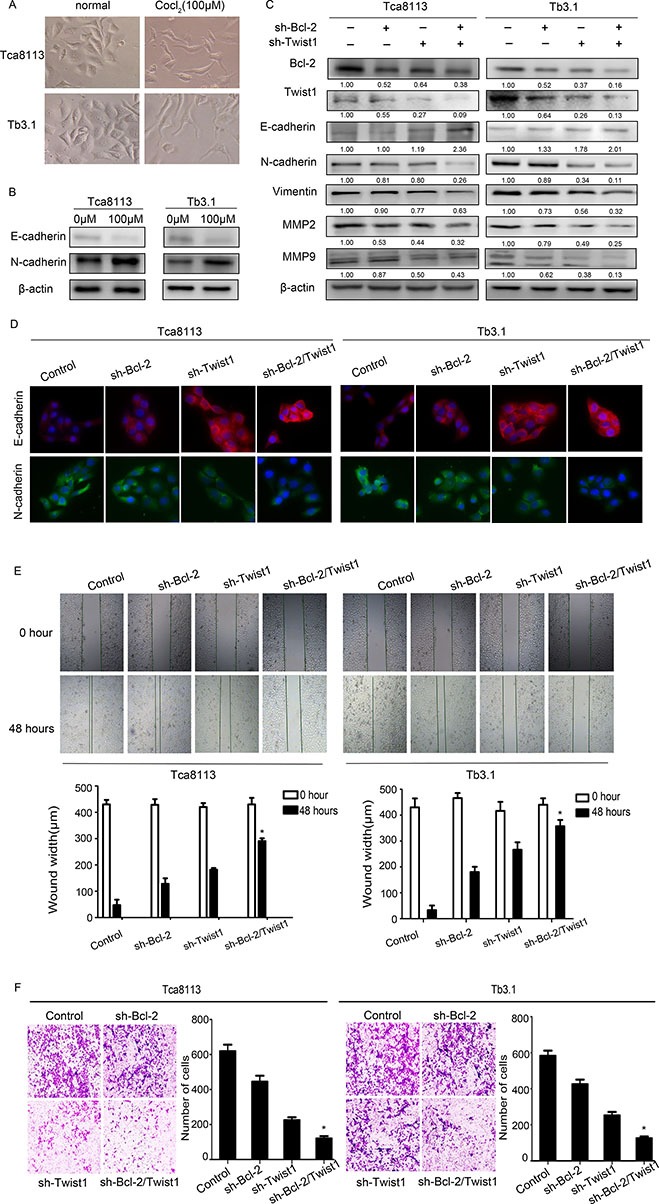
Coexpressed Bcl-2 and Twist1 contribute to tumor cell EMT **(A)** and **(B)** Hypoxia induced EMT in OSCC cell lines. **(C)** and **(D)** Detection of relative protein in Tca8113 and Tb3.1 cells after silencing Bcl-2 or Twist1 by Western blot and immunofluorescence. N-cadherin, Vimentin and MMP2/9 were down-regulated, the E-cadherin level was upregulated. **(E)** and **(F)** Scratch and transwell assays were performed to determine the motility and invasion, capacity of the sh-Bcl-2/Twist1 group was significantly decreased.

In the present study, OSCC EMT was induced by CoCl_2_ (Figure 4A and 4B). We silenced Bcl-2 and Twist1, separately or together, and examined the EMT marker expression. The downexpression of Bcl-2 alone reduced Twist1 and epithelial markers. Similar results were observed for sliencing Twist1. The above observation was rescued by simultaneously blocking Bcl-2 and Twist1 expression, resulting in EMT features, such as elevated E-cadherin expression, whereas the expression of N-cadherin and Vimentin was obviously attenuated and tumor EMT was notably reversed (Figure 4C). This result was further validated by immunofluorescence staining (Figure 4D). In addition, MMP-2 and MMP-9 were inhibited, which is in line with the down- regulation of Bcl-2 or Twist1, especially in the Bcl-2/Twist1 combined treatment group (Figure 4C).

The scratch and transwell assays were performed to determine the motility and invasion in these cells. The relative distance was greatest in the sh-Bcl-2/Twist1 groups compared to the other three groups (migration relative distance (μm) in Tca8113: control: 382.7 ± 47.6, sh-Bcl-2: 300 ± 72.2, sh-Twist1: 238.3 ± 15.8, sh-Bcl-2/Twist1: 139.3 ± 48.7, *p* = 0.001; in Tb3.1: control: 396.7 ± 24.9, sh-Bcl-2: 286 ± 4.3, sh-Twist1: 150 ± 32.7, sh-Bcl-2/Twist1: 82.7 ± 31.8, *p* = 0.000; Figure 4E). Furthermore, an invasion assay showed that the number of invadingcells in the control group was significantly higher than that in the sh-Bcl-2/Twist1 group (invading cells in Tca8113: control: 612 ± 52.4, sh-Bcl-2: 445 ± 47.7, sh-Twist1: 225.3 ± 23.3, sh-Bcl-2/Twist1: 120.3 ± 18.8, *p* = 0.000; in Tb3.1: control: 582.7 ± 41.5, sh-Bcl-2: 425.7 ± 35.5, sh-Twist1: 252.3 ± 27.1, sh-Bcl-2/Twist1: 125.7 ± 14.4, *p* = 0.000. Figure 4F). The Bcl-2/Twist1 complex, compared to either Bcl-2 or Twist1 alone, is more efficient in promoting EMT and tumor metastasis in OSCC.

### Bcl-2/Twist1 complex depletion inhibited Tca8113 xenograft tumor growth and the EMT process

**Figure 5:**
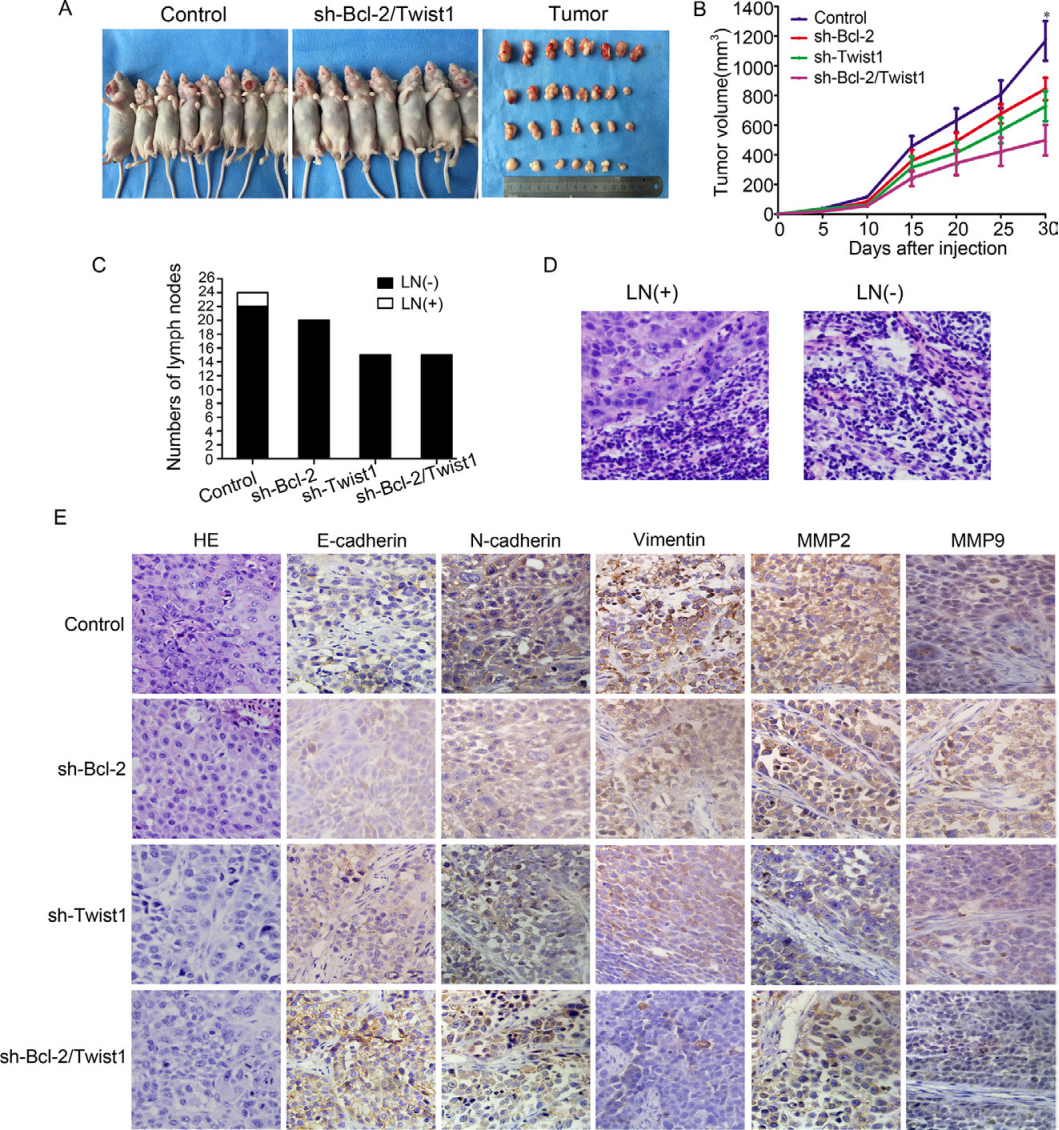
Silencing of Bcl-2/Twist1 inhibited growth of Tca8113 xenograft tumor in the mouth floor *in vivo* **(A)** and **(B)** Exhaustion of Bcl-2/Twist1 inhibited tumor growth, *p* = 0.001. **(C)** In the control group, 2 of 24 lymph nodes had tumor metastasis whereas 20 lymph nodes of in the sh-Bcl-2 group and 16 in both the sh-Twist1 and sh-Bcl-2/Twist1 groups were negative. **(D)** HE staining was used to examine lymph node metastasis. **(E)** Immunohistochemical staining of Bcl-2, Twist1, E-cadherin, N-cadherin, Vimentin, and MMP2/9 in murine tumors.

We developed a Tca8113 xenograft tumor model by injecting tumor cells in the mouth floor to confirm the consequence of silencing Bcl-2/Twist1 *in vivo*. Knockdown of the Bcl-2/Twist1 complex inhibited the growth of Tca8113 xenograft tumors (Figure 5A and 5B). H&E staining showed that two lymph nodes in the control group were diagnosed with metastatic lesions, whereasthe sh-Bcl-2 and sh-Bcl-2/Twist1 groups had metastatic lesions in all examined lymph nodes (Figure 5C and 5D). We speculate that combined silence of Bcl-2/Twist1 inhibits the expression of EMT-markers N-cadherin and Vimentin, thus leading to decreased MMP-2/9 expression and cell invasion (Figure 5E). The results of *in vivo* studies suggest that Bcl-2/Twist1 depletion can, to a great extent, inhibit tumor growth and metastasis.

## DISCUSSION

In tumorigenesis, enhanced Bcl-2 expression has been defined as a central factor that plays a key role in dysregulated programmed cell death pathways by suppressing apoptosis and prolonging cell survival [18–20]. Although much is known about the anti-apoptotic ability of Bcl-2, little information is available about its functions in other cellular processes. To date, there is limited experimental evidence suggestings the involvement of Bcl-2 in tumor EMT progression. Juan An et al. [21] revealed that in breast cancer, constitutive Bcl-2 expression affects the expression of the genes that determine cellular behaviors, such as the loss of epithelial cellular marker E-cadherin and gain of mesenchymal N-cadherin. The shift mechanism in Bcl-2 transfected cells indicated that Bcl-2 expression may induce typical EMT. In our study, we demonstrate knocking down expression of Bcl-2 initiated cascade response and changed a typical EMT process in OSCC. Additionally, depletion of Bcl-2 significantly suppressed cell migration and invasion. Twist1 played a decisive role in tumor EMT, similarly, in our research, silencing of Twist1 resulted in a distinguished MET process. Interestingly, all results showed that the mesenchymal phenotype, migration and invasion of the two cell lines were remarkably inhibited by inhibiting the expression of both Bcl-2 and Twist. In other words, the present study supported that the Bcl-2/Twist1 complex, compared to either Bcl-2 or Twist1, is more efficient in promoting EMT and tumor metastasis in OSCC.

The tumor microenvironment is a fundamental condition to malignancy, and hypoxia is a key factor in this process. Tumor cells respond to hypoxia,which results in the downregulation of epithelial genes expression and in the activation of genes that help define the mesenchymal phenotype. Mesenchymal-like epithelial tumors obtains the capacity to increase cell protrusions and motility degrade extracellular matrix (ECM) proteins, thereby enabling invasive behavior [11, 22, 23]. Hypoxia, which is a common characteristic of solid tumors, has been reported to reactivate EMT through hypoxia-induced factor-1 alpha (HIF-1α ) in several tumor models [24–27]. HIF-1a directly regulates Twist1 , and then transactivates EMT-related genes such as, E-cadherin, Vimentin, and N-cadherin to mediate EMT [28–30]. In the present study, hypoxia and hypoxia-induced EMT were triggered by CoCl_2_. Twist1 up-regulation peaked 12 and 24 hours after hypoxia in two cell lines. Interestingly, Bcl-2 also exhibited an expression peak and trend similar to those of Twist1. Then, we explored the interaction between Bcl-2 and Twist1 using Co-IP, and the evidence indicates that the two proteins were physiologically bound. However, hypoxia can facilitate and amplify even active Bcl-2 to form a complex with Twist1. The synergistic reaction of the Bcl-2/Twist1 heterodimer is more powerful in stimulating the transcription of multiple downstream target genes than Bcl-2 or Twist1 alone. We also found that in hypoxia stress, of Bcl-2 and Twist1 in OSCC were abundantly located in the nucleus. It has been reported that Twist1 has a nuclear localization signal that induces Twist1 to enter the nucleus and act as a transcription factor [31], but itsunderlying regulation remains unclear. Sun et al. [13] found that specific amino acids within Bcl-2 and Twist1 are involved in the binding of two proteins, and that they form a novel functional complex, an activated transcript complex, which promotes the nuclear transport of Twist1, tumor cell metastasis and vasculogenic mimicry. Additionally, the nuclear expression of Bcl-2 and Twist1 is correlated with poor survival in hepatocellular carcinoma. From present results, we safely posit a potential mechanism that over-expressed Bcl-2 binds to Twist1 and that the formation of the Bcl-2/Twist1 complex promotes the transport of two factors into nucleus, which synergistically promotes the transcription of downstream target genes and leading to a cascade of changes in cell phenotype remodeling, migration, invasion and tumor growth.

This is a study exploring the role of Bcl-2 and Twist1 in OSCC EMT. Our findings demonstrated the importance of a Bcl-2/Twist1 functional complex in OSCC EMT and metastasis, that affects the patients’ prognosis. For a long time, Bcl-2 has been considered a key mitochondrial membrane protein in intrinsic (mitochondrial) apoptotic pathways, and its role in EMT was first demonstrated in OSCC. Although further study is need to define the detailed mechanisms, our results indicate that there is an interaction between Bcl-2 and Twist1 in OSCC. In summary, our study raised a novel mechanism for developing of EMT in the presence of Bcl-2 and cancer progression mediated by a Bcl-2/Twist1 functional complex, which may provide a new therapeutic target for OSCC treatment.

## MATERIALS AND METHODS

### Tissue samples

A total of 82 patients with OSCC were includedin the study, which was approved by the Tianjin Medical University Cancer Institute & Hospital Ethic Committee. All patients had received radical resection of the tumor and neck lymph node dissection at Tianjin Medical University Cancer Institute & Hospital between January 2006 and June 2008. In addition, 24 paraneoplastic tissues were used as a control. Of the 82 OSCC patients, 56 were males (68.3%) and 26 were females (31.7%). Their age ranged from 36 to 78 years, with a median age of 57.5 years. Clinico-pathological variables, such as the gender, age, tumor site, pathologic grading, clinical staging, and lymph node status, were obtained from the medical records, as shown in Table 1. Survival information of the 82 patients was obtained from the visits by letter or telephone. Informed consent was obtained from each subject.

### Immunohistochemistry (IHC)

For IHC staining, paraffin-embedded tissue slides (4μm thick) were deparaffinized, rehydrated, and incubated with primary antibodies overnight at 4°C. The following antibodies were used: Bcl-2, Twist1, N-cadherin, matrix metalloproteina-2/9 (MMP-2/9) (Abcam, Cambridge, MA, USA), E-cadherin, and Vimentin (Cell Signaling Technology, USA). Then, the slides were incubated with biotin-labeled secondary antibody (Maxin Bio Corp., Fujian, China) for 1 h at room temperature and incubated with diaminobenzidine (Zhongshan Bio Corp., Beijing, China). After, counterstaining with hematoxylin, slides were dehydrated with different concentrations of alcohol and soaked in xylene. Then, they were mounted with neutral balsam and visualized using a light microscope. Ten representative fields at 400× magnification per slide were observed. The results were assessed by measuring both the staining intensity (0 = negative, 1 = light staining, 2 = moderate staining, and 3 = intense staining) and the number of positive cells ( 0 means 0–5% positive cells; 1, 6–25% positive cells; 2, 26–50% positive cells, and 3, 51–100% positive cells). The scores for the intensity and percentage of positive cells were multiplied to detemine a weighted score for each case. A score of 0–3indicated low expression (−), and scores of 4–9 indicated high expression (+).

### Cell culture and transfection

The human Tca8113 OSCC cell line was purchased from the Chinese Academy of Medical Sciences Institute of Basic Medical Sciences and Tb3.1 was kindly donated by Shanghai Ninth People's Hospital. The cell lines were grown in RPMI-1640 (Hyclone, USA) supplemented with 10% fetal bovine serum (FBS, Hyclone, USA). Cells were incubated in a humidified atmosphere of 5% CO2 and 95% air at 37°C. LV-shRNA-Bcl2 and LV-shRNA-Twist1 were purchased from Genepharm (China). A LV-shRNA empty plasmid was used as a negative control. A total of 300 ng/ml plasmid was transfected with Lipofectamine 2000 (Invitrogen, Carlsbad, CA, USA) according to the manufacturer's instructions. 100 mM/ml Cocl2 was used to mimic hypoxia microenvironment.

### RNA extraction and qPCR

Total RNA was extracted using Trizol (Life technology, USA) reagent according to the manufacturer's protocol and reverse transcribed into cDNA using M-MLV Reverse Transcriptase (Life technology, USA). RT-qPCR was performed using the Step One Plus system (Applied Biosystems, USA). The Bcl-2 primer were “TTGTTCAAACGGGATTCACA” (Forward) and “GAGCAAGTGCAGCCACAATA” (Reverse), and the primer of Twist1 primer were “GCAAGAAGTCGAGCGAAGAT” (Forward) and “GCTCTGCAGCTCCTCGAA” (Reverse) (Sangon Biotech, China). The RT-qPCR procedure was performed under the following conditions: 30 sec at 95°C followed by 40 cycles of 5 sec at 95°C and 34 sec at 60°C. All Bcl-2 and Twist1 expression data were normalized to GAPDH. The Ct value of each target gene was normalized against the Ct value of the reference gene.

### Coimmunoprecipitation (Co-IP)

Cell lysates with 1000 μg of protein prepared from Tca8113 and Tb3.1 cells were cleaned with protein A/G beads before they were subjected to Co-IP using 1 μg of Twist1(Mouse genus) or Bcl-2(Rabbit genus) antibody. An equal level of IgG was used as the negative control. Immunocomplexes were denatured by boiling in a sodium dodecyl sulfate-polyacrylamide gel electrophoresis (SDS-PAGE) sample buffer, and were separated in 10% SDS-PAGE gels for western blot analysis using Twist1 and Bcl-2 antibodies.

### Western blotting

After the cells were transfected and cultured for 48 hours, RIPA lysis buffer was used to split cells (Millipore, MA, USA). A Nuclear and Cytoplasmic Extraction Reagents kit (Beyotime Biotechnology, China) was used according to the manufacturer's instructions. The details were as follows: Cell plasma protein extraction reagent A was added to the cell precipitate, vortexed for 5 seconds and palced in an ice bath for 15 minutes. Then, cell plasma protein extraction reagent B was added; the mixture was vortexed for 5 seconds and centrifuged (12000 g; 5 min). The supernatant that was plasma protein. Cell nuclear protein extraction reagent was added tp the residual precipitation and samples were vortexed for 30 seconds and centrifuged (16000 g; 10 min). The supernatant was nuclear protein. The protein concentrations of the supernatants were determined using the bicinchoninic acid protein assay. Typically, 30 μg of protein was then separated by SDS-polyacrylamide gel electrophoresis. The gel was transferred onto a PVDF film with 300 mA at 4°C for 1.5 h, using a wet electroblotting system (Bio-Rad, Hercules, CA, USA). The membranes were blocked in blocking buffer (5% nonfat dry milk, 0.1% Tween in PBS) for 1.5 h and then incubated with primary antibodies overnight and horseradish peroxidase-conjugated secondary antibodies (Zhongshan Bio Corp.). Immunoreactive protein bands were visualized using an enhanced chemi-luminescence detection system (SuperSignal ECL Kit, Thermo Scientific) and subsequent exposure of the membrane to Hyperfilm (Thermo Scientific). The same membrane was probed with murine anti-β-actin or Histone H3 (Abmart) as a loading control.

### Cell invasion and wound healing assays

Cell invasion assays were performed using transwell membranes coated with Matrigel (BD Biosciences). Transfected cells were plated at a density of 5 × 10^4^ cells per well. The lower chamber was filled with 20% FBS. After 48 h, the noninvasive cells in the upper chamber wereremoved with cotton swabs, and invasive cells were fixed with 95% ethanol for 15 min and then stained with 0.5% crystal violet. Cells penetrating through the polyethylene terephthalate membrane were counted in ten representative microscopic fields. In wound healing assays, cell motility was assessed by measuring the movement of cells into a scarp. The speed of wound closure was monitored after 48 h by measuring the ratio of the distance of the wound distance from 0 h.

### Immunofluorescence (IF)

Cells adhered to the cover slip and grew until 50%–60% confluence. The cells were then fixed for 10 min with cold methanol, sealed with fetal calf serum, and incubated with primary antibodies overnight at 4°C. Fluorescein isothiocyanate-labeled secondary antibody (Invitrogen, USA) was added at 37°C for 2 h. Diamidino-phenyl-indole reagent was used to stain the cell nuclei, and the cells were visualized using a FV-1000 laser scanning confocal microscope (Olympus, Tokyo, Japan).

### Tca8113 cells xenograft tumor model

All animal experimental protocols were approved by Tianjin Medical University Animal Care and Use Committee. The BALB/c 5-week-old female nude mice were maintained in SPF grade (Vital River Laboratories, China). All mice were implanted in the mouth floor with 1.0 × 10^7^ Tca8113 cells and different Bcl-2 or Twist1 expression levels. The tumor volume was measured with a caliper every 3 days using a formula (volume = (long diameter × short diameter^2^)/2). After 30 days, the mice were sacrificed and xenograft tumors were removed for formalin fixation and the preparation of paraffin-embedded sections.

### H&E staining

At the end of the 30-day observation period, the mice bearing xenograft tumors were sacrificed and tumor tissues were removed for formalin fixation and the preparation of paraffin-embedded sections. H&E staining was performed to observe the tumor pathological changes under a microscope.

### Statistical analysis

Data were analyzed using SPSS 18.0. The interrelationship of Bcl-2, Twist1 and EMT related proteins expression with clinic-pathologic variables was analyzed using the χ^2^ or Fisher exact test. Spearman's correlation test was used to analyze the correlation between Bcl-2 and Twist1 with the EMT-related proteins. Kaplan-Meier and time series tests (log-rank test) were used for univariate survival analysis. A *P value* less than 0.05 was considered statistically significant.
